# GWAS Central: an expanding resource for finding and visualising genotype and phenotype data from genome-wide association studies

**DOI:** 10.1093/nar/gkac1017

**Published:** 2022-11-09

**Authors:** Tim Beck, Thomas Rowlands, Tom Shorter, Anthony J Brookes

**Affiliations:** Department of Genetics and Genome Biology, University of Leicester, Leicester, LE1 7RH, UK; Health Data Research UK (HDR UK), London, UK; Department of Genetics and Genome Biology, University of Leicester, Leicester, LE1 7RH, UK; Department of Genetics and Genome Biology, University of Leicester, Leicester, LE1 7RH, UK; Department of Genetics and Genome Biology, University of Leicester, Leicester, LE1 7RH, UK; Health Data Research UK (HDR UK), London, UK

## Abstract

The GWAS Central resource gathers and curates extensive summary-level genome-wide association study (GWAS) data and puts a range of user-friendly but powerful website tools for the comparison and visualisation of GWAS data at the fingertips of researchers. Through our continued efforts to harmonise and import data received from GWAS authors and consortia, and data sets actively collected from public sources, the database now contains over 72.5 million P-values for over 5000 studies testing over 7.4 million unique genetic markers investigating over 1700 unique phenotypes. Here, we describe an update to integrate this extensive data collection with mouse disease model data to support insights into the functional impact of human genetic variation. GWAS Central has expanded to include mouse gene–phenotype associations observed during mouse gene knockout screens. To allow similar cross-species phenotypes to be compared, terms from mammalian and human phenotype ontologies have been mapped. New interactive interfaces to find, correlate and view human and mouse genotype–phenotype associations are included in the website toolkit. Additionally, the integrated browser for interrogating multiple association data sets has been updated and a GA4GH Beacon API endpoint has been added for discovering variants tested in GWAS. The GWAS Central resource is accessible at https://www.gwascentral.org/.

## INTRODUCTION

Genome-wide association studies (GWAS) provide insights into the cause of disease by testing genetic variants across multiple genomes to find the variants that are statistically associated with complex traits and disease phenotypes. GWAS results have a range of applications such as predicting disease risk, understanding the genetic architecture of phenotypes, and estimating their heritability. As sequencing costs reduce and GWAS population sample sizes regularly exceed more than 1 million participants, GWAS are increasingly using whole exome sequencing and whole genome sequencing to allow the identification of rare variants which may explain missing heritability in complex traits (1). Additionally, reviews have shown that efficacy in drug development can be improved by targeting GWAS disease risk genes (2,3). GWAS also has a role in gaining insight into a phenotype's underlying biology and characterising functional elements in the mammalian genome (4).

An effective approach for improving our understanding of the functional landscape of the mammalian genome is the comparison of genetic variants and associated phenotypes across species. The mouse is a prevalent mammalian disease model and is routinely used to determine the causal relationship between gene and disease, and to predict the functional impact of human genetic variation (5). The International Mouse Phenotyping Consortium (IMPC) has the aim of characterising the function of every protein coding gene by undertaking genome- and phenome-wide phenotyping of mouse lines (6). Mouse cohorts that have a null mutation in a unique protein coding gene are screened using a standardised and comprehensive phenotyping pipeline that measures over 500 phenotyping parameters (7). Phenotypic observations are curated with the Mammalian Phenotype Ontology (MPO) (8). Potential disease models have been identified from integrations of IMPC gene–phenotype associations with Online Mendelian Inheritance in Man (OMIM) (9) genetic diseases. These data integrations are used to determine if a mouse gene is an orthologue of a disease-causing human gene (10). A phenotype similarity approach is used to detect IMPC mouse strains that have a strong phenotype overlap for a given disease (11). Aligning IMPC gene–phenotype associations with GWAS would provide a transformational basis to help identify causative genes among GWAS data by providing functional evidence to support the involvement of a candidate gene in an overlapping phenotype. Additionally, given that the majority of the variants identified by GWAS are non-coding (12) and thought to regulate gene expression, comparing human loci and mouse genes that share overlapping phenotypes could provide insights into candidate causal genes by linking non-coding variants to their target genes.

GWAS databases such as the NHGRI-EBI GWAS Catalog (13), GWASdb (14) and PheGenI (15) provide open access to limited amounts of summary-level GWAS data. A common feature of these repositories is that their complete content, or a large portion of it, is restricted to marker signals that exceed predefined *P*-value thresholds. GWAS Central imposes no such restrictions and provides bench scientists and bioinformaticians with access to a uniquely comprehensive collection of GWAS summary-level data. A toolkit of user-friendly search interfaces, browsers and graphical displays enable real-time interrogation and visualisation of customisable views of the data along with information about the tested markers. For SNP markers, this information includes chromosome position, flanking sequences, and alleles. Researchers can discover and compare data sets of interest from the perspective of genes, genome regions, phenotypes, or disease. Given the risk of identifying individuals from pooled summary-level data (16), GWAS Central limits the display of risk alleles per SNP to ensure research study participants are not re-identified.

Since our previous report on the database (17), GWAS Central has attained ELIXIR-UK Node service status. ELIXIR is the European research infrastructure for life sciences data and has national nodes that contribute well used services that are representative of the bioinformatics efforts within the country. During an open selection process, the UK Node of ELIXIR evaluates resources against a set of published criteria (18), including interoperability with other resources, evidence of community outreach and high community adoption/usage. It was through community engagement with human and mouse genetics researchers that we identified the requirements for the updates that we describe here. We have integrated IMPC gene knockout data with summary-level GWAS, provided ontology term mappings between mouse and human ontologies and added new phenotype and genome browsers to compare orthologous phenotypes and genomic loci. Additionally, we have updated the APIs available to support programmatic discovery of GWAS data and updated the integrated browser, for comparing multiple GWAS data sets across regions of the genome, by introducing new comparative variant data tracks.

## MATERIALS AND METHODS

### GWAS data updates

GWAS Central continues to collect and curate GWAS summary-level association data and study metadata from publication supplementary materials, submissions from GWAS authors and consortia, and imports from other GWAS databases including the NHGRI-EBI GWAS Catalog. The phenotypes investigated in each study are annotated using ontology terms from the NLM’s Medical Subject Headings (MeSH) controlled vocabulary and Human Phenotype Ontology (HPO) (19). If an exact ontology term does not exist for a phenotype, the annotation is recorded in the database as a non-exact match. An updated version of MeSH is released every year that includes additional terms (HPO is updated more regularly). Non-exact matches are re-evaluated annually using the latest versions of MeSH and HPO and more precise terms are applied if they have become available.

A goal of GWAS Central is to allow multiple GWAS to be compared. To support this, an integrated browser tool allows up to 16 association data sets to be correlated and visually interrogated. Following feedback from GWAS Central users about the ‘region’ view of the browser, which allows regions of the genome to be examined in increasing resolution down to individual nucleotide level, we added new tracks to support the comparison of GWAS signals alongside DisGeNET (20) and 1000 Genomes Project (21) variants. The DisGeNET variant-disease association data file, and the 1000 Genomes Project variants file available from the International Genome Sample Resource (IGSR) (22), were transformed into the General Feature Format (GFF) required by the region browser. Due to the high density of variants from the 1000 Genomes project, non-GWAS markers were hidden.

### Integration of mouse genotype–phenotype data

We processed the IMPC open data files to extract gene–phenotype associations and procedure metadata. The use of different phenotype ontologies between the human and mouse communities has been identified as a major barrier to computational interpretation of cross-species (in this case mouse and human) genotype–phenotype associations (23). The IMPC dataset incorporates a total of 547 MPO terms, which include terms used to annotate genes and intermediate terms between annotations and the ontology root. We have previously described our method for mapping exact terms in MeSH and HPO (24). Extending this work, we initially evaluated public sources of ontology mappings for MPO to MeSH and MPO to HPO from NCBO BioPortal (25) and EMBL-EBI OxO, however these were incomplete. Therefore, we developed an algorithm for mapping MPO, MeSH and HPO terms based on the Lexical OWL Ontology Matcher (LOOM) method (26). LOOM removes delimiters and compares terms and synonyms between ontologies for matches with an allowance of one character difference for strings larger than four characters in length. We added a rule set to reduce false positive results, such as preventing the interchange of ‘h’ and ‘w’ at the start of a string, thus eliminating the false positive match of ‘weight’ and ‘height’. MeSH defines traits and MPO defines phenotypes (traits with values), so we normalised MPO phenotypes to traits before we applied the mapping algorithm. For example, we removed the string ‘abnormal’ from the start of MPO terms, hence the MPO term ‘abnormal lung compliance’ can be mapped to the MeSH term ‘Lung Compliance’. The mappings were manually evaluated, and additional mappings included where the semantic similarity between terms had not been detected using our lexical similarity method, for example, the MPO term ‘enlarged heart’ was manually mapped to the MeSH term ‘Cardiomegaly’ (41% of mappings were manually assigned). We previously described the role of graph databases in storing phenotype ontology mappings within the GWAS Central system architecture (17). We imported MPO and mappings to MeSH and HPO into the ontology graph and made these accessible to the interfaces via an API. Human orthologues of the IMPC mouse genes, and their coordinates mapped to genome assembly GRCh37, were retrieved by using Ensembl BioMart to query the human genes and mouse genes datasets. The interfaces for comparing human and mouse association data were developed using the React JavaScript library and the region view browser uses JBrowse 2 (27).

## RESULTS

### Interrogating GWAS data

**Figure 1.**
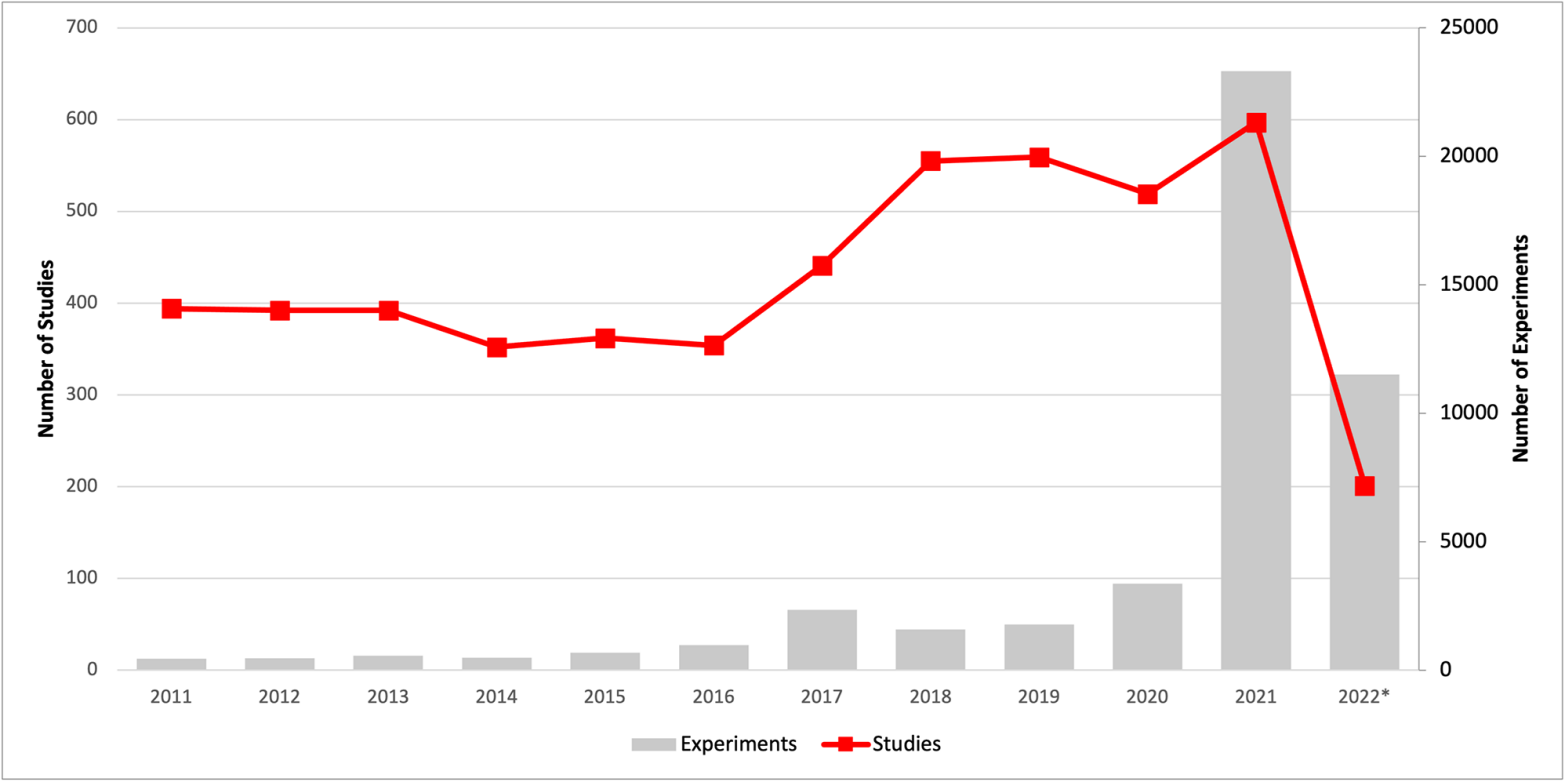
Studies and experiments added to the GWAS Central database since 2011. There was a large increase in the number of experiments in 2021, with 2022 being comparable (* includes studies up to July 2022).

To date, we have gathered 72 538 116 *P*-value associations for 5026 studies testing 7 453 618 unique genetic markers investigating 1773 unique MeSH phenotype descriptions. Figure 1 shows the general trend of increasing study and experiment (a sub-study examining one phenotype) numbers year-on-year since 2011. Recently, there has been a large increase in the number of experiments reported, caused by individual studies that investigate associations to -omic phenotypes such as the hundreds of experiments reported in lipidome (28), metabolome (29) and microbiome (30) related GWAS.

**Figure 2.**
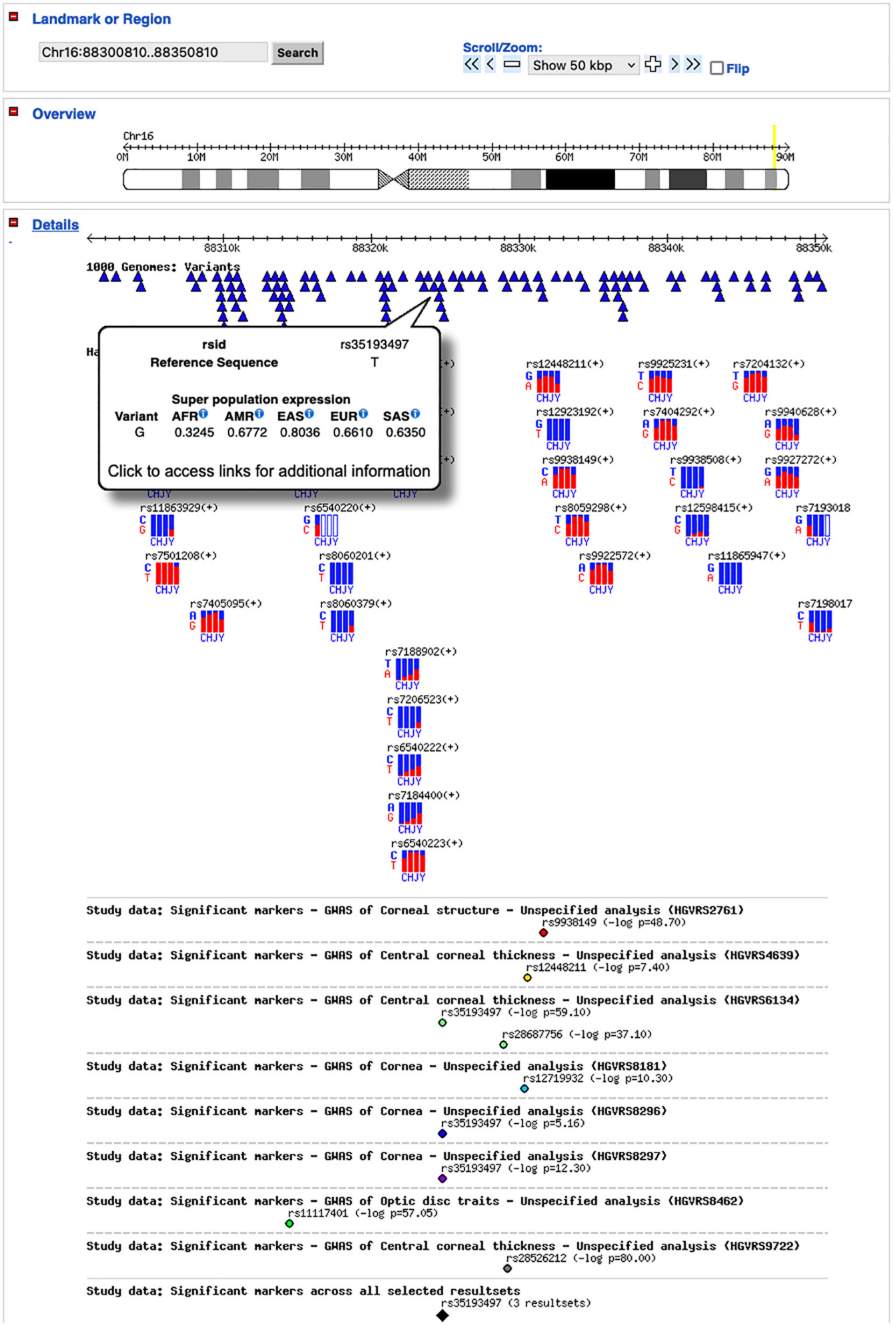
GWAS Central region browser example. Markers from eight data sets investigating corneal morphology phenotypes are compared in a 50 kb region on chromosome 16. Three data sets have significant associations with SNP rs35193497. Hovering over rs35193497 in the 1000 Genomes track displays the allelic expression in super populations. Tracks can be displayed or hidden as required (some tracks have been hidden). Super population abbreviations: AFR – African, AMR – American, EAS – East Asian, EUR – European, SAS – South Asian.

The GWAS Central browser allows interesting signals from multiple correlated association data sets to be examined in detail by switching between the ‘genome’ view of all chromosomes and the higher resolution ‘region’ view. The displays allow P-value thresholds to be applied, therefore presenting only those associations a researcher deems significant. The region view browser provides context to GWAS signals by supporting the use of annotation tracks displaying genes, HGMD variants, HapMap SNPs and linkage disequilibrium maps. The DisGeNET and 1000 Genomes Project data are presented as integrated browser tracks. Selecting a variant of interest within a track will display links to the respective external resource. Furthermore, the 1000 Genome Project track displays allelic expression rates within the five IGSR super populations (Figure 2).

We previously described the GWAS Central REST-based web services for exporting study reports and performing remote searches of markers, genes and regions, marker association results, phenotypes and studies (17). Documentation is provided to describe the site-specific query parameters interpreted by these web services. To further the discovery and reuse of GWAS data sets, variants tested in GWAS can be queried from a Global Alliance for Genomics and Health (GA4GH) Beacon v1.0 API endpoint (https://beacon.gwascentral.org/). The Beacon endpoint provides a standardised interface to discover GWAS variants alongside our site-specific web services.

### Comparing human and mouse genotype–phenotype associations

New interfaces for comparing human and mouse association data are found in the ‘Homology’ tab on the website. In summary; (i) the phenotype searches retrieve GWAS and mouse genes associated to phenotypes of interest, (ii) for selected phenotypes the genome view aligns human orthologs of IMPC mouse genes and GWAS variants against the human genome, (iii) selected genome regions can be viewed in greater resolution using the region view browser. The displays allow *P*-value thresholds to be set independently for the human and mouse associations.

**Figure 3.**
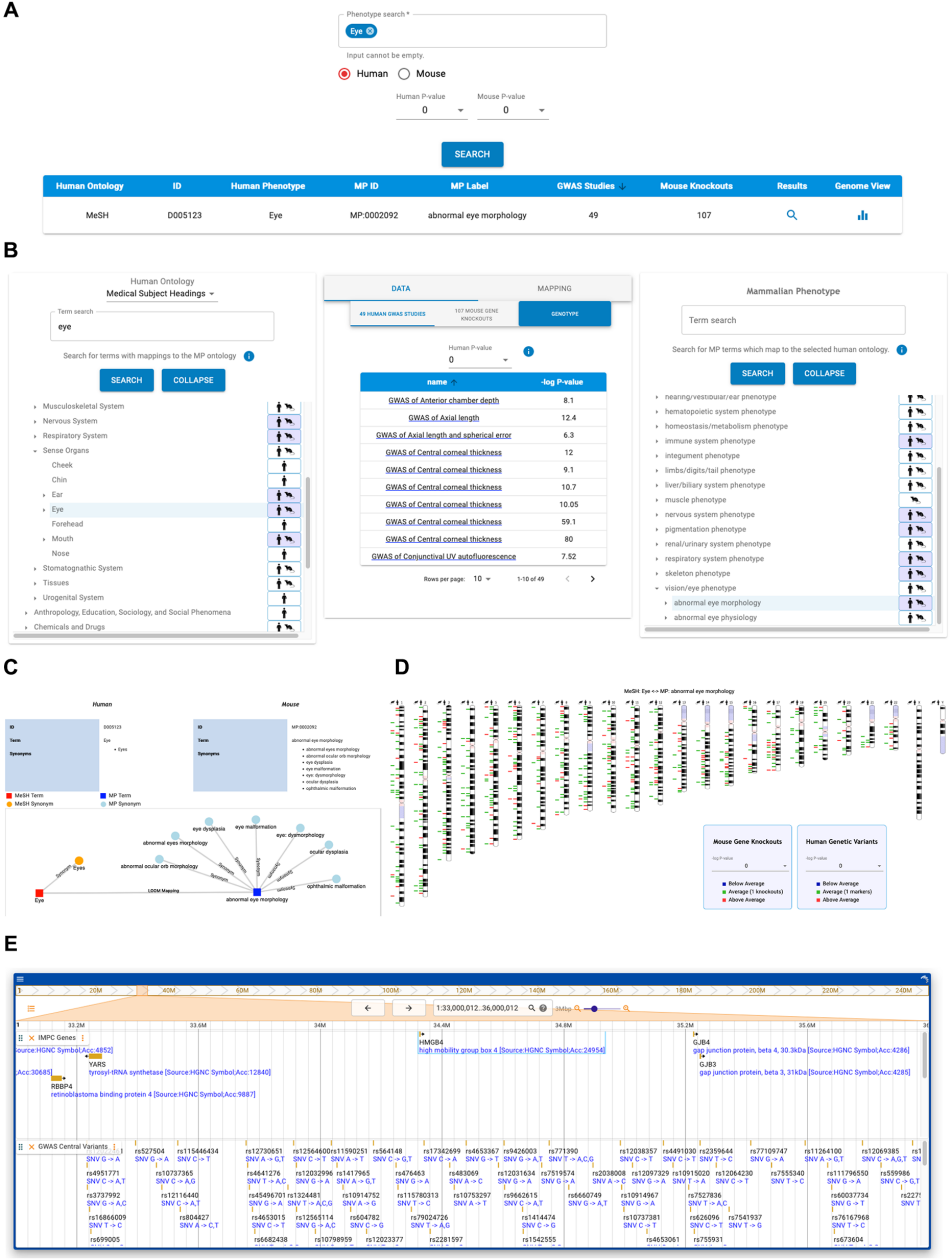
Interfaces for exploring integrated GWAS and mouse gene data. (**A**) The results from the text search displays term mappings and the number of GWAS studies and mouse gene knockouts matching the query term(s). (**B**) The ontology hierarchy comparison interface displays which terms are associated with human and mouse data sets. A search using one ontology will retrieve records annotated to the mapped term. In this example, a MeSH GWAS search for ‘eye’ phenotypes also retrieves mouse genes annotated to ‘abnormal eye morphology’. (**C**) The ontology mapping graphic presents the source of the term mapping. (**D**) The heatmap alongside each chromosome indicates the number of mouse genes (first column) and GWAS markers (second column) per 3 Mb bin having a *P*-value that passes a tunable significance threshold. (**E**) A dynamic region-level browser, where scale and position may be tailored to one's preferences, presents optional tracks for individual GWAS marker associations and IMPC mouse genes.

Phenotypes can be searched by ontology terms or by browsing ontology hierarchies. The phenotype text search interface can be used from the human or mouse perspective, querying by MeSH and HPO, or MPO terms and synonyms, respectively. A search will return the number of GWAS Central studies and IMPC gene knockouts annotated to the search and mapped terms (Figure 3A). A report of the results includes links to the matched GWAS Central studies, IMPC gene knockouts, and associated IMPC procedure metadata. The text search interface allows multiple terms to be searched in one query using the logical ‘OR’ operator between terms. The ontology hierarchy interface allows side-by-side navigation and comparison of the human and mouse ontology trees (Figure 3B). The trees include terms that are used in either GWAS Central or IMPC annotations or result from mappings between the two. Each term in the tree has an icon that denotes the type of data annotated with that term and the type of mapping. The icon symbol indicates if the term will retrieve mouse or human records only, or mouse and human records via a term mapping. The icon background is coloured blue to indicate a direct mapping, where records from the other species are retrieved due to a mapping to that term. A white icon background indicates inferred mapping, where records from the other species are retrieved due to mappings to descendent terms in the hierarchy. A data report is displayed in the centre panel (Figure 3B) and contains links to GWAS and IMPC for matched records. If an ontology term is mapped, selecting that term will refocus the opposite ontology on to the mapped term and the ontology mapping graphic is displayed. This graphic displays the source of the mapping (LOOM or manual) and which terms or synonyms were matched (Figure 3C). Results from phenotype searches can be further explored in genome-wide displays of association findings in the genome view. A heatmap of human genetic markers and mouse gene knockouts associated with the phenotype is plotted against human chromosomes (Figure 3D). From these views users can select a region of interest to navigate to a higher-resolution region view browser (Figure 3E). The region view browser displays a track of human orthologues of the mouse genes and a track of GWAS variants which can be browsed to the resolution of individual nucleotides.

## DISCUSSION

We have expanded GWAS Central with mouse gene–phenotype association data to support genome-wide translational research involving mouse models of human disease and to enable new insights into the functional effects of human genetic variation. However, most SNPs associated with disease phenotypes are in non-coding regions, so further utility will be achieved by integrating GWAS data with mouse genetic studies that investigate phenotypes associated with variants in genome regions beyond the ∼5% that encodes for proteins. We will extend our mouse data integrations to include QTL studies to enable the comparison of loci associated with similar phenotypes. Mouse genetic studies have the potential to validate GWAS findings (31), and comparisons of human and mouse genomic regions associated to disease traits have been used to demonstrate the capabilities of GWAS summary-level data to support the clinical relevance of mouse genetic screens (32).

The GA4GH Beacon API is designed to query human genomics data sets for the presence or absence of variants. Version 1 of the protocol standardised the ‘do you have this variant?’ request received by a resource and the ‘yes’ or ‘no’ response. Networks of Beacons demonstrated federated genomic data querying where a single query is sent to multiple genomics resources, and they rapidly respond with an answer. The GWAS Central Beacon API v1.0 endpoint allows researchers to query GWAS Central in parallel with other genomics resources for variants of interest. If the markers have been tested in at least one GWAS, and so are shown as being present in GWAS Central, further searches can be undertaken using the GWAS Central REST web service or the website to find more details about the variant, including all phenotypes the variant is associated with. Version 2 of the Beacon API extends the scope of supported queries and responses (33). Beacon v2.0 requests can include phenotypes and responses can include record details and ‘handovers’ to the native data exchange format. Our future plans for GWAS Central include providing a Beacon v2.0 API endpoint to support more sophisticated queries for GWAS markers associated with phenotypes. We will provide handovers to relevant GWAS Central reports for more information, including study metadata that describes how individual-level data may be requested for GWAS that have tested variants of interest.

There is a trend for increasing numbers of GWAS to contain experiments investigating high numbers of phenotypes (Figure 1). To support the GWAS Central database curators with annotating an expanding quantity of data, we have developed regular expression-based methods for automatically matching free-text phenotype descriptions with ontology terms. GWAS Central and other databases that curate publications face the challenge of scalable curation as the amount of data in the scientific literature, tables, and supplementary materials, grows. Text mining systems have been used to extract biological entities and the relations between them from the literature, including gene-disease interactions (34,35) and variant-disease associations (36). However, current systems are not without limitations. Most approaches focus on text mining in publication abstracts and there is no public text mining application, to our knowledge, capable of extracting genotype–phenotype associations from publication tables. The broad variability in table structure makes them difficult to mine automatically. We have developed the Automated pipeline for Consistent Outputs from Research Publications (Auto-CORPus) text processing tool that converts publication full-text and tables to standardised machine-interpretable formats that can be analysed by text mining algorithms (37). In a collaboration with ELIXIR researchers, we are building Auto-CORPus into a text mining workflow to extract GWAS associations from the scientific literature at scale. The workflow will be used by GWAS Central (and other resources) to populate the database and support the efforts of database curators.

## DATA AVAILABILITY

GWAS Central is available from https://www.gwascentral.org. The Beacon API endpoint is at https://beacon.gwascentral.org. A complete study or 1000 markers and associated data per download is provided by GWAS Mart (BioMart based system) at https://mart.gwascentral.org. Larger data downloads are available to researchers upon agreement with GWAS Central's data sharing policy at https://help.gwascentral.org/data/data-sharing-statement.
